# Massive pericardial effusion causing cardiac tamponade accompanied by elevated CA-125 and thoracic lymphadenopathy in sarcoidosis: a case report

**DOI:** 10.1016/j.ijscr.2020.06.037

**Published:** 2020-06-13

**Authors:** Taalaibek Kudaiberdiev, Elmira Tukusheva, Zhanybek Gaibyldaev, Gulnaz Tursunbekova, Zhunus Kadyraliev, Irina Akhmedova, Nurjan Tulopbergenov, Emil Muraliev

**Affiliations:** Scientific Research Institute of Heart Surgery and Organ Transplantation, Bishkek, Kyrgyzstan

**Keywords:** CA, carbohydrate antigen, CT, computed tomography, LV, left ventricle, LVPW, left ventricular posterior wall, RA, right atrium, RV, right ventricle, Pericardial effusion, Tamponade, Sarcoidosis, CA-125, Mediastinal lymphoadenopathy, Pericardial lymphoadenopathy, Case report

## Abstract

**Introduction:**

Pericardial effusion and cardiac tamponade are rare manifestations of cardiac sarcoidosis. This is a first case report that describes a patient with severe pericardial effusion and signs of cardiac tamponade with elevated carbohydrate antigen 125 (CA-125) levels, enlarged pericardial (PLN) and mediastinal lymph nodes (MLN), histologically confirmed as sarcoidosis.

**Presentation of case:**

A 51-year-old female patient was admitted with complaints of sickness, shortness of breath on minimal exertion, swelling in lower extremities, and heaviness in right upper abdomen. Patient had diminished heart sounds, peripheral edema and hepatomegaly. She had elevated CA-125 level without gynecologic pathology. There were QRS alternation on ECG and water-bottle configuration on chest-X-ray, severe pericardial effusion, and signs of cardiac tamponade on echocardiography. CT demonstrated massive pericardial effusion, pericardial mass and enlargement of anterior MLN.

The patient underwent pericardial drainage with removal of 850 mL of pericardial fluid and excision of enlarged PLN. Histological examination of PLN revealed non-caseating epithelioid cell granulomas. The diagnosis of cardiac sarcoidosis was established. Patient was discharged and 6-month follow-up was uneventful.

**Discussion:**

There are no reports on association of pericardial effusion, with increased CA-125 level in sarcoidosis, as we established in our patient. Our case is notable by incidental finding of enlarged PLN, mimicking pericardial mass and mediastinal lymphoadenopathy on CT, further confirmed by histological examination of PLN specimen as cardiac sarcoidosis.

**Conclusion:**

It should be kept in mind that sarcoidosis may present as massive pericardial effusion, with signs of tamponade and pericardial lymphoadenopathy mimicking pericardial mass, mediastinal lymphoadenopathy and elevated CA-125, mimicking malignancy.

## Highlights

• Sarcoidosis may present as pericardial effusion with thoracic lymphoadenopathy.
• Elevated CA-125 levels may accompany pericardial effusion in cardiac sarcoidosis.
• Cardiac tamponade may complicate pericardial effusion in sarcoidosis.

## 1 Introduction

Sarcoidosis is a systemic granulomatous disease of unknown etiology, characterized by the formation of epithelioid granulomas in lungs, peripheral lymph nodes, skin, eyes and liver [1]. Cardiac sarcoidosis is a rare entity, and occurs in 2–7% of patients, while latent course is much more frequent [2]. It occasionally occurs in the absence of pulmonary or systemic involvement [2,3].

Cardiac sarcoidosis can involve the myocardium, endocardium and pericardium, with a variety of clinical manifestations, with the most frequent being arrhythmia and conduction disturbances, and heart failure [[4], [5], [6], [7]].

Pericardial effusion is detected on echocardiography in 10–21% of patients with pulmonary or systemic sarcoidosis, even in the absence of symptoms [3]. Isolated pericardial involvement is infrequent and may be manifested as pericardial effusion, constrictive pericarditis and very rarely tamponade [3,4,[8], [9], [10], [11]]. Confirming the diagnosis of cardiac sarcoidosis is a difficult task [6]. The clinical manifestations of cardiac sarcoidosis with pericardial lesions are similar to those of pericardial diseases of other etiologies [3,4]. Cardiac sarcoidosis is accompanied by high mortality and is one the main causes of death in sarcoidosis [12].

We describe a first case of cardiac sarcoidosis manifested by symptomatic severe pericardial effusion, with signs of cardiac tamponade, and accompanied by increased carbohydrate antigen-125 (CA-125) levels and mediastinal and pericardial lymph node enlargement, verified by histological examination of pericardial lymph node specimen and successfully treated by pericardial drainage and excision of lymph node at Scientific Research Institute of Heart Surgery and Organs Transplantation.

The work has been reported in line with the SCARE criteria [13].

### 1.1 Presentation of case

A 51-year-old female patient was admitted with complaints of sickness, severe shortness of breath on minimal exertion, moderate swelling in lower extremities, and heaviness in right upper abdomen. The patient felt sick during one month before admission when shortness of breath and swelling of legs had appeared. Before admission, the patient received therapy with non-steroidal antiinflammatory drugs, antibiotic and diuretic. However, due to lack of positive response to treatment after two weeks, the patient was referred to our cardiac surgery clinic with diagnosis of pericardial effusion of unknown etiology and bilateral pleural effusion. Patient had no history of gynecologic diseases. Informed consent was obtained from patient for all diagnostic and treatment procedures.

General physical examination was unremarkable except for peripheral edema. There was hepatomegaly on palpation and diminished lung sounds and weakened heart sounds on auscultation. The patient had NYHA functional class III.

Laboratory results (Table 1) demonstrated mild lymphocytosis (lymphocyte count of 42%), negative antistreptolysin O, C-reactive protein and rheumatoid factor tests, negative tests for markers of viral hepatitis, human immunodeficiency virus, cytomegalovirus and herpes virus. However, the high values of tumor marker CA- 125 (201 IU/L) were detected, while other tumor markers (Table 1) were negative.

**Table 1:** Laboratory blood tests data of a patient before and after treatment.

Parameters	Before treatment	After treatment
Hemoglobin	137 g/dl	150 g/dL
Red blood cell count	4.5 × 10^12^/L	5 × 10^12^/L
Hematocrit	41%	45%
Platelets	216 × 10^9^/L	240 × 10^9^/L
White blood cell count	6.6 × 10^9^/L	7.9 × 10^9^/L
Stab	5%	4%
Neutrophils	44%	65%
Eosinophils	3%	4%
Basophils	0%	0%
Monocytes	6%	7%
Lymphocytes	42% (normal value 19–37%)	20%
Sedimentation rate	11 mm/h	18 mm/h
Human immunodeficiency virus test	negative	–
Virus hepatitis test	negative	–
Wasserman reaction	negative	–
Antistreptolysin O	200 IU/mL (normal value 0–200 IU/mL)	–
Rheumatoid factor	Negative	–
C- reactive protein	Negative	–
Carbohydrate antigen - 125	201 IU/L (normal value < 35 IU/L)	–
Cytokeratin 19 fragment- CYFRA 21−1	1.5 ng/mL (normal value 0–3.3 ng/mL)	–
Human chorionic gonadotropin	1.37 IU/l (normal value <2 IU/l),	–
Carbohydrate antigen 15 – 3	5.5 IU/l (normal value <30 IU/L)	–
Carcinoembryonic antigen	0.97 ng/mL (normal value <5 ng/mL)	–
Antibody titer to cytomegalovirus	230 U/mL	–
Antibody titer to herpes virus	33.7 U/mL	–

ECG showed sinus tachycardia (90 beats/min) and low voltage QRS. Chest X-ray displayed a water-bottle configuration of the heart with an increased cardiothoracic ratio and a small amount of fluid in both pleural spaces.

**Fig. 1:**
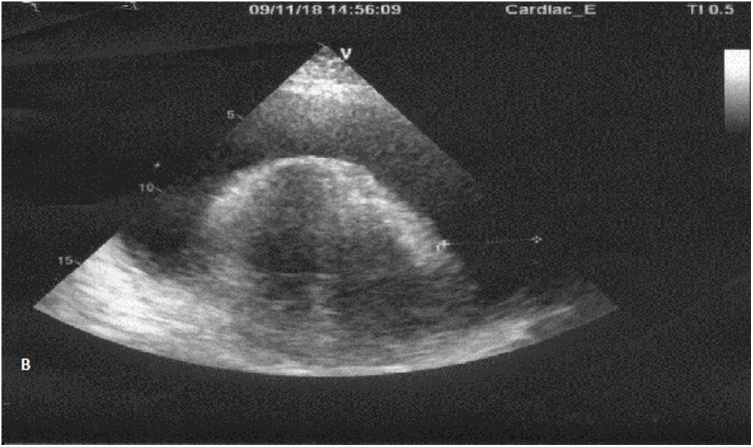
Echocardiography image of a massive pericardial effusion.

Echocardiography (Table 2, Fig. 1) demonstrated large pericardial effusion, with maximum width of fluid up to 38 mm, 24 mm, 35 mm and 22 mm behind LVPW, LV apex, RV and RA, respectively, and collapses of the RA and RV. Additional echocardiographic findings were minimal mitral, tricuspid and pulmonary regurgitations, LV diastolic dysfunction and preserved LV ejection fraction (68%) with no hypokinesia zones.

**Table 2:** Echocardiography data before and after treatment.

Parameters	On admission	After pericardial drainage
Aorta	Unremarkable, ascending part diameter 24 mm	–
Descending aorta	Unremarkable	–
Aortic valve	Tricuspid, pressure gradient - 5 mm Hg	–
Mitral valve	Minimal regurgitation, maximal pressure gradient - 3 mm Hg	–
Tricuspid valve	Minimal regurgitation maximal, pressure gradient - 3 mm Hg	Minimal regurgitation
Pulmonary artery	Normal size	–
Pulmonary valve	Minimal regurgitation, pressure gradient - 5 mm Hg	–
Left atrium	33 mm	36 mm
Right atrium	Not expanded	Normal
Left ventricle (LV):		
LV end-diastolic size	45 mm	39 mm
LV end-systolic size	28 mm	22 mm
LV ejection fraction	68%	76%
The interventricular septum	9 mm	–
The posterior wall of the LV	9 mm	–
LV regional contractility	No hypokinesia zones	–
LV diastolic function	E/A – abnormal pattern	Normal
Right ventricle	22 mm	–
Right ventricular free wall thickness	4 mm	–
Systolic pulmonary artery pressure	35–40 mm Hg	–
Pericardium:	large effusion	Minimal effusion
LV posterior wall	37 mm	0.8 mm
LV apex	24 mm	0 mm
Right ventricular free wall	35 mm	0 mm
Right atrium	22 mm	0 mm
	Small collapse of right atrium and right ventricle	No signs of collapse
Atrial septum	intact	–
Ventricular septum	intact	–
Pleural cavities:		No effusion
The left echonegative space	12 mm	
The right echonegative space	34 mm	
Vena cava inferior	22 mm, collapses on inspiration less than 50%

**Fig. 2:**
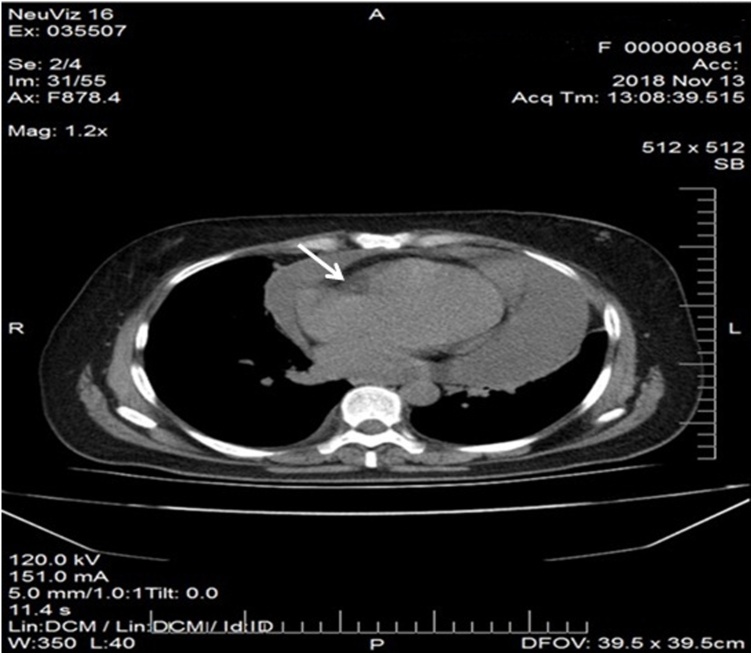
Computed tomography image of large pericardial effusion and pericardial mass in pericardial fat tissue (enlargement of pericardial lymph node?) (arrow).

**Fig. 3:**
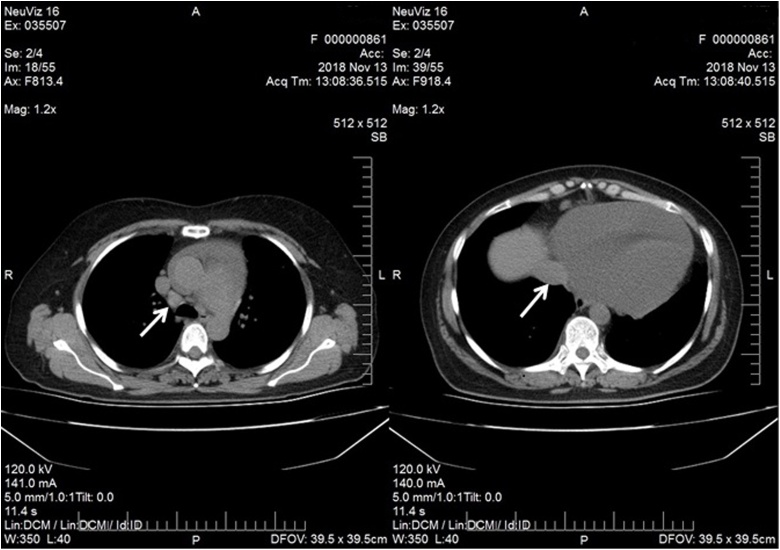
Computed tomography images of lymph nodes enlargement (arrows) in anterior mediastinum.

Computed tomography (CT) revealed large pericardial effusion, (Fig. 2) with localized mass in pericardial fat (possibly lymph node), small pleural effusion and enlarged lymph nodes in anterior mediastinal compartment (Fig. 3).

Due to clinical and imaging signs of symptomatic large pericardial effusion, with signs of tamponade and presence of pericardial mass (possible lymph node), the patient underwent drainage of pericardial cavity and excision of pericardial lymph node through subxyphoid approach under endotracheal anesthesia using standard technique. During the procedure, pericardium was found to be thickened and a total of 850 mL of serous-sanguineous fluid was evacuated. Part of pericardium was excised for bacteriological and histological examination.

There was an enlarged lymph node in pericardium, which was dissected and sent for histological examination. Central venous pressure reduced from 120 mm to 30 mm after procedure.

**Fig. 4:**
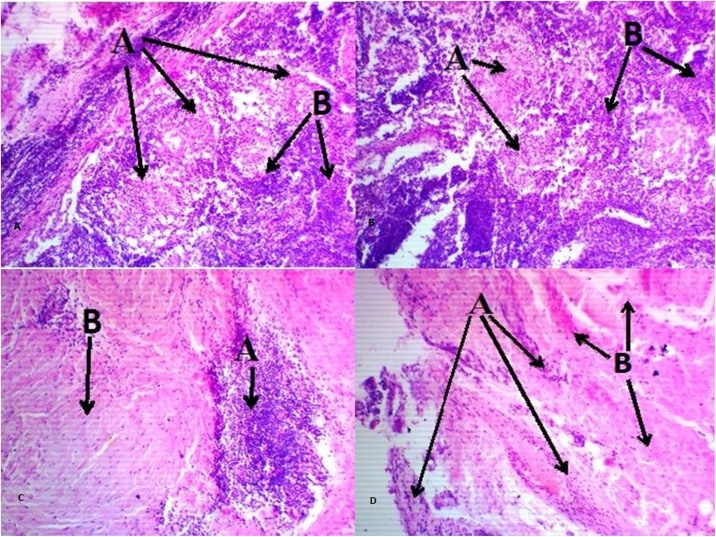
Micropreparations of lymph nodes No. 17,133,366 (edge part). A) A- giant multi-core cells, B- healthy tissue; B) Granulomas with accumulation of epithelioid cells of macrophages on the periphery (giant multi-core cells) - A- giant multi-core cells, B- healthy tissue; C) Micropreparations of lymph nodes No. 17,133,366 (central part); Granuloma with accumulation of epithelioid cells in the center macrophages - A - focal lymphocytic infiltration. B- healthy tissue D) Pericardial micropreparations №27,133,366 of focal lymphocytic infiltration - A - focal lymphocytic infiltration. B - healthy tissue.

Cytological examination showed no atypical cells in the pericardial fluid. Histological examination of lymph node revealed non-caseating granulomas with accumulation of epithelioid cells (giant multi-core cells) (Fig. 4A) in the center (Fig. 4B) and along the periphery (Fig. 4C); pericardial specimen demonstrated focal pericardial lymphocytic infiltration (Fig. 4D), the findings associated with sarcoidosis.

After pericardial drainage, patient’s condition had improved, there was only minimal fluid posteriorly (0.8 mm) on echocardiogram (Table 2), and minimal pleural effusion on chest X-ray. She was discharged on 8thday, and her follow-up at 6 months after treatment was uneventful without complaints.

## 2 Discussion

We presented as a rare case of cardiac sarcoidosis in a female patient manifested as massive pericardial effusion, with signs of cardiac tamponade and elevated CA-125 tumor marker, mediastinal and pericardial lymphoadenopathy, diagnosed using histological analysis of pericardial lymph node specimen and successfully treated by pericardial drainage and excision of enlarged pericardial lymph node.

Cardiac sarcoidosis without extracardiac manifestations is seen in young and middle-aged women, and is manifested by arrhythmias and conduction disturbances and heart failure [6,7,14]. Isolated large pericardial effusion, and cardiac tamponade are rare in sarcoidosis [3,4,8,10,11]. Our case was remarkable due to signs of large pericardial effusion, on ECG (low voltage QRS), chest X-ray (water-bottle configuration), CT, echocardiogram and signs of tamponade on echocardiography (RA and RV collapse).

The search for specific etiology of pericardial effusion, in our patient was negative according to laboratory results. The presence of high level of CA-125, known as a tumor marker in ovarian carcinoma [15], posed a diagnostic dilemma. However, our patient had no associated gynecologic pathology. CA-125 is a glycoprotein with embryonic epithelial source including not only fallopian tubes, endometrium, endocervix, but also pleura, pericardium, and peritoneum [15].

Elevated level of CA- 125 in sarcoidosis was associated with pleural effusion [16] and peritoneal involvement [15].

Presence of pleural effusion in our patient corroborates with previously reported case [15]. However, there are no reports on association of pericardial effusion, with CA-125 level in sarcoidosis, as we established in our patient. Thus CA-125, as glycoprotein responsible for origin of pericardium from embryonic source can be increased in cases of sarcoidosis with pericardial effusion.

Our case is notable by absence of clinical signs of cardiac sarcoidosis with incidental finding of enlarged lymph node of pericardium, mimicking pericardial mass and mediastinal lymphoadenopathy on CT, further confirmed by histological examination of pericardial lymph node specimen.

The diagnosis of sarcoidosis is usually established by clinicoradiological findings of lymphoadenopathy with or without pulmonary involvement, or pulmonary only involvement, supported by histological findings of non-caseating epithelioid cell granulomas [1]. The lymphoadenopathy in sarcoidosis is usually bilateral hilar and right paratracheal, while isolated mediastinal lymphoadenopathy is a rare finding [17]. Hilar lymphoadenopathy is usually established by chest radiography, while CT is superior for diagnosis of mediastinal lymphoadenopathy and parenchymal involvement [17].

Mediastinal lymphoadenopathy was detected by CT in 88% of histologically proven cardiac sarcoidosis cases manifesting as dilated cardiomyopathy and in all cases with arrhythmias, undergoing radiofrequency ablation and ICD placement [7,18]. The case with cardiac tamponade and mediastinal lymphoadenopathy was also reported [11].

Our patient had signs of mediastinal lymphoadenopathy on CT, but our case was distinguishable by presence of pericardial lymphoadenopathy on CT, mimicking pericardial mass and confirmed intraoperatively.

For histological diagnosis of cardiac sarcoidosis, endomyocardial biopsy may be used, though biopsies of mediastinal lymphoadenopathy guided by CT or PET imaging have been shown to be accurate [6,19].

We could not find studies with pericardial lymphoadenopathy and cardiac sarcoidosis proven by histological analysis of pericardial lymph node. Examination of specimen of pericardial lymph node demonstrated findings associated with sarcoidosis. There was only one report on presence of multiple nodules in pericardium of a patient with cardiac tamponade, in whom biopsy of pericardium demonstrated non-caseating granulomas [8].

We undertook open-heart surgery due to presence of pericardial mass on CT and large pericardial effusion. The intervention revealed the enlarged pericardial lymph node suspected by CT, which was excised and sent together with pericardial specimens for histological examination that revealed non-caseating granulomas specific for cardiac sarcoidosis.

Treatment of sarcoidosis with signs of large pericardial effusion, usually includes pericardiocentesis, drainage and corticosteroids therapy [4,5,11,20]. In very rare cases of constrictive pericarditis, pericardectomy is indicated [9]. Treatment of cardiac sarcoidosis is based on use of corticosteroids, but immunosuppressive therapy may be required 5,11]. Therapy of cardiac sarcoidosis with involvement of myocardium and arrhythmias and conduction disturbances are ablation of ventricular tachycardia source, ICD implantation for secondary prevention of sudden cardiac death and pacing [7,14].

## 3 Conclusion

This is the first case reporting increased CA-125 levels associated with pericardial effusion with signs of tamponade, pericardial and mediastinal lymphoadenopathy in cardiac sarcoidosis, established by histological analysis of pericardial lymph node specimen. One should keep in mind that cardiac sarcoidosis may present as massive pericardial effusion, with signs of tamponade and pericardial lymphoadenopathy mimicking pericardial mass, mediastinal lymphoadenopathy and elevated CA-125, mimicking malignancy.

## Funding

There is no funding received for case report.

## Ethical approval

No ethical approval is required for case report.

## Consent

Written informed consent was obtained from patient for all procedures, for publication of case report and all accompanying images.

## Author contribution

(1) the conception and design of the study, or acquisition of data, or analysis and interpretation of data, (2) drafting the article or revising it critically for important intellectual content, (3) final approval of the version to be submitted.

All authors equally contributed to management of patient and preparation of case report, the conception and design of the study, acquisition of data, analysis and interpretation of data, drafting the article or revising it critically for important intellectual content, final approval of the version to be submitted.

## Authorship

T. K., E.T., Zh.G., G.T., Zh.K., I.A., N.T. equally contributed to preparation of case report.

## Registration of research studies

No registration is required for research study.

## Guarantor

Taalaibek Kudaiberdiev.

## Provenance and peer review

Not commissioned, externally peer-reviewed.

## Declaration of Competing Interest

There is no conflict of interest to declare.
